# Supplementary material for: Impaired SorLA maturation and trafficking as a new mechanism for SORL1 missense variants in Alzheimer disease
Source: Acta Neuropathol Commun. 2021 Dec 18;9:196. doi: 10.1186/s40478-021-01294-4 (PMC8684260; doi:10.1186/s40478-021-01294-4)
Supplement: Supplementary file 1 — Additional file 1.: Supplementary figures. [file 40478_2021_1294_MOESM1_ESM.pdf]

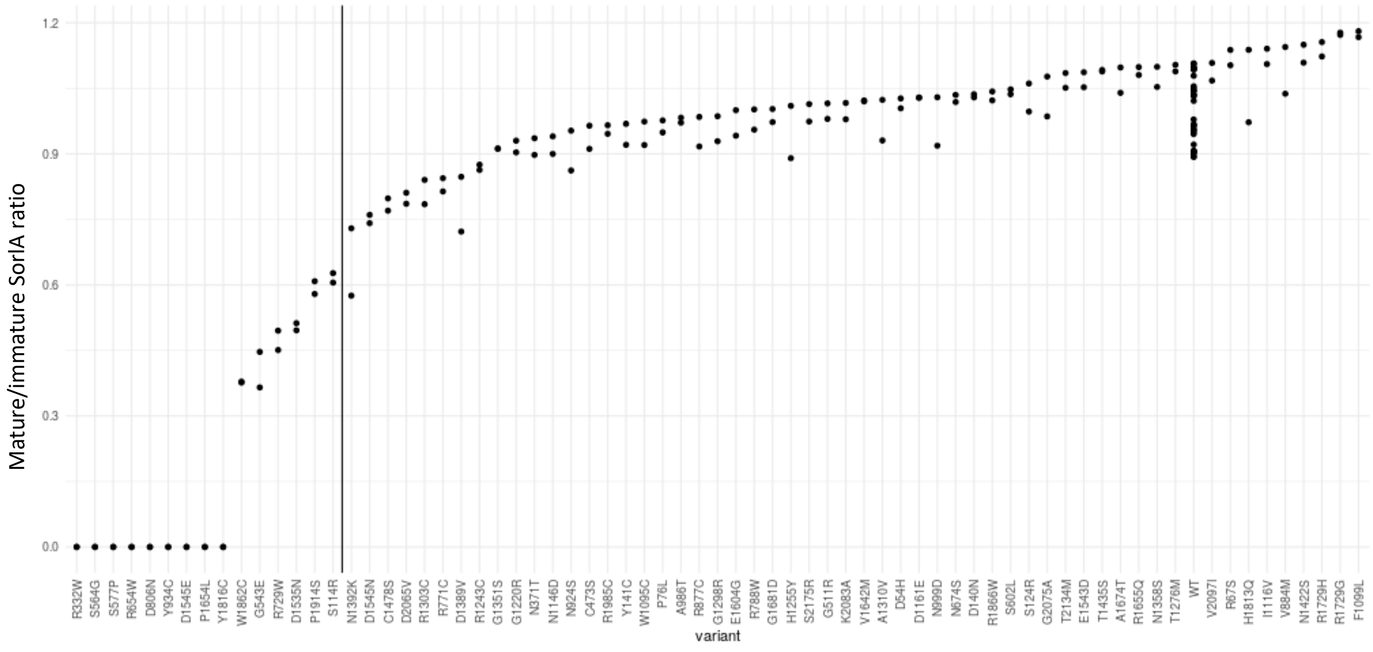
Mature/immature SorlA ratio
1.2
0.9
0.6
0.3
0.0
R332W
S564G
S577P
R654W
D806N
Y934C
D1545E
P1654L
Y1816C
W1862C
G543E
R729W
D1535N
P1914S
S114R
N1392K
D1545N
C1478S
D2065V
R1303C
R771C
D1389V
R1243C
G1351S
G1220R
N371T
N1146D
N924S
C473S
R1985C
Y141C
W1095C
P76L
A986T
R877C
G1298R
E1604G
R788W
G1681D
H1255Y
S2175R
G511R
K2083A
V1642M
A1310V
D54H
D1161E
N999D
N674S
D140N
R1866W
S602L
S124R
G2075A
T2134M
E1543D
T1435S
A1674T
R1655Q
N1358S
T1276M
WT
V2097I
R67S
H1813Q
I1116V
V884M
N1422S
R1729H
R1729G
F1099L
variant

Fig.S1

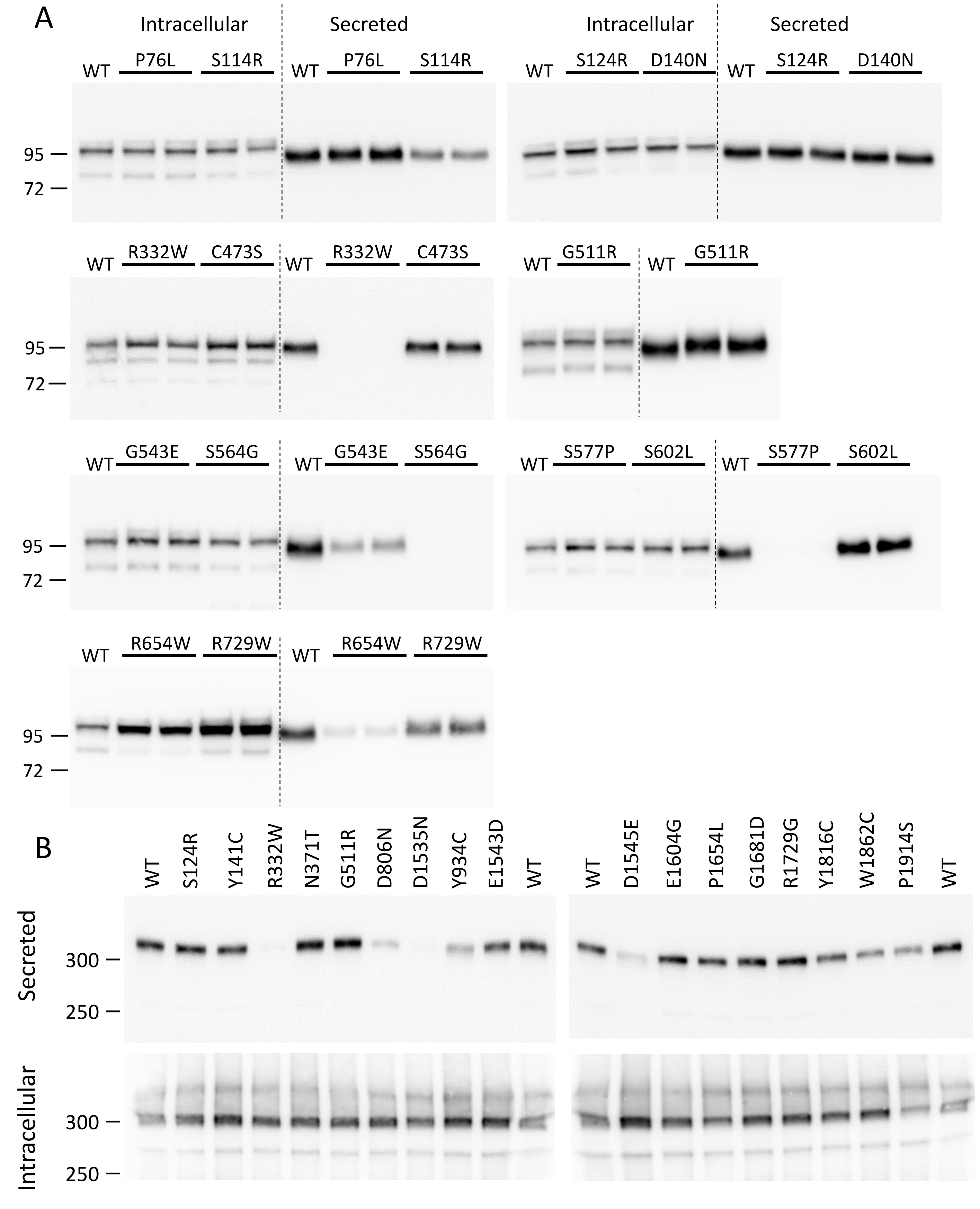
A
Intracellular
Secreted
WT
P76L
S114R
S124R
D140N
R332W
C473S
G511R
G543E
S564G
S577P
S602L
R654W
R729W
95
72
B
S124R
Y141C
N371T
D806N
D1535N
Y934C
E1543D
D1545E
E1604G
P1654L
G1681D
R1729G
Y1816C
W1862C
P1914S
300
250

Fig.S2

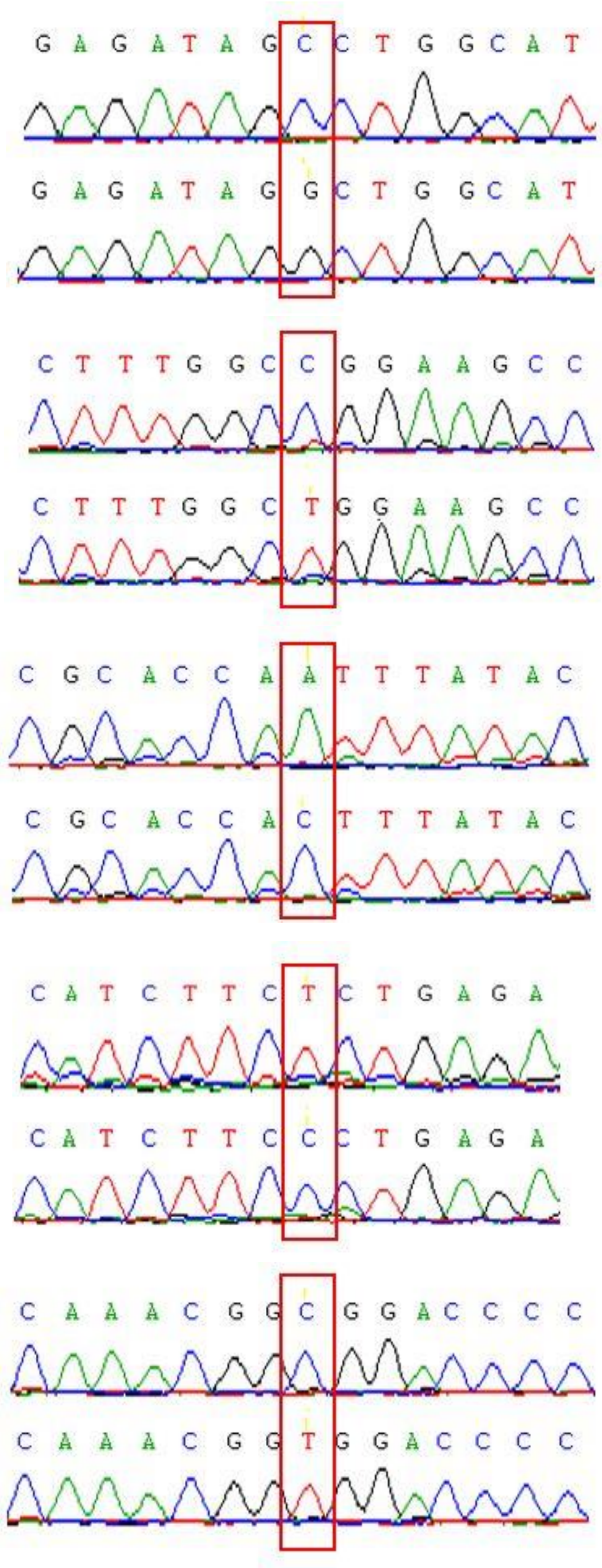

c.372C>G
p.(Ser124Arg)

c.994C>T
p.(Arg332Trp)

c.1112A>C
p.(Asn371Thr)

c.1729T>C
p.(Ser577Pro)

c.1960C>T
p.(Arg654Trp)

Fig.S3

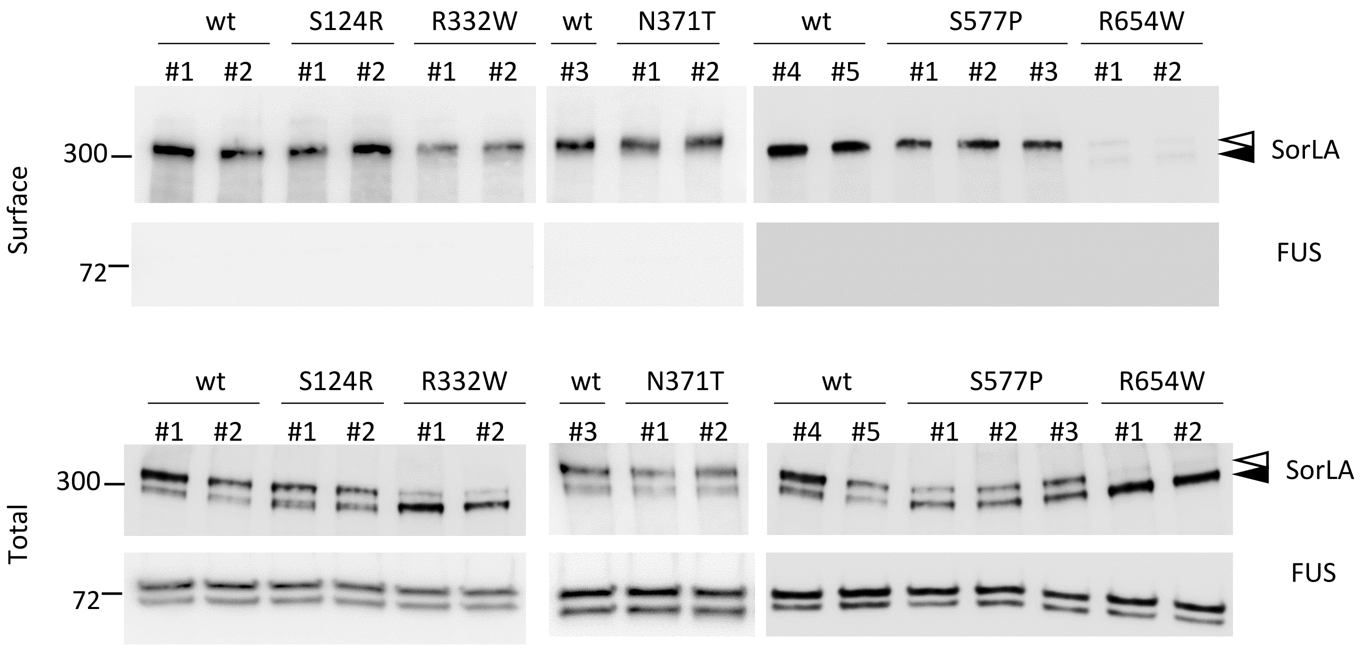

wt
S124R
R332W
wt
N371T
wt
S577P
R654W
#1 #2 #1 #2 #1 #2 #3 #1 #2 #4 #5 #1 #2 #3 #1 #2
300
SorLA
Surface
72
FUS
wt
S124R
R332W
wt
N371T
wt
S577P
R654W
#1 #2 #1 #2 #1 #2 #3 #1 #2 #4 #5 #1 #2 #3 #1 #2
300
SorLA
Total
72
FUS

Fig.S4

A

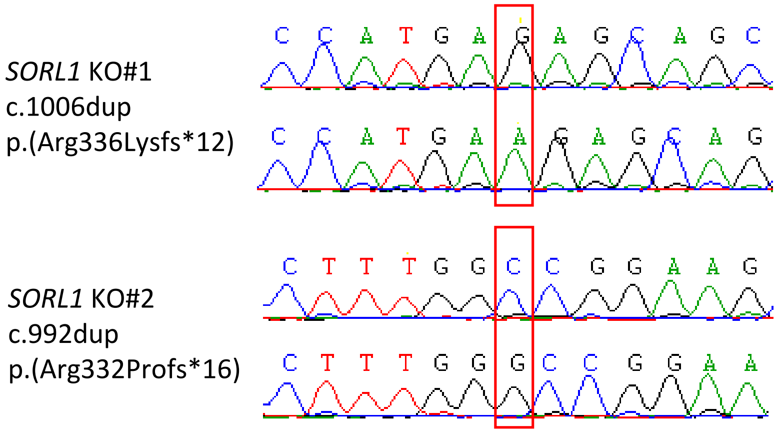
SORL1 KO#1
c.1006dup
p.(Arg336Lysfs*12)
SORL1 KO#2
c.992dup
p.(Arg332Profs*16)

B

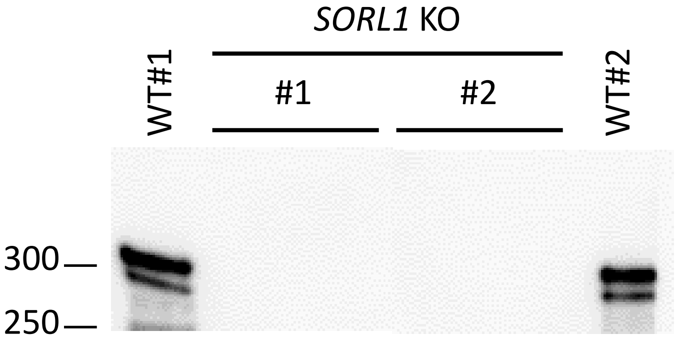
SORL1 KO
WT#1
#1
#2
WT#2
300
250

Fig.S5

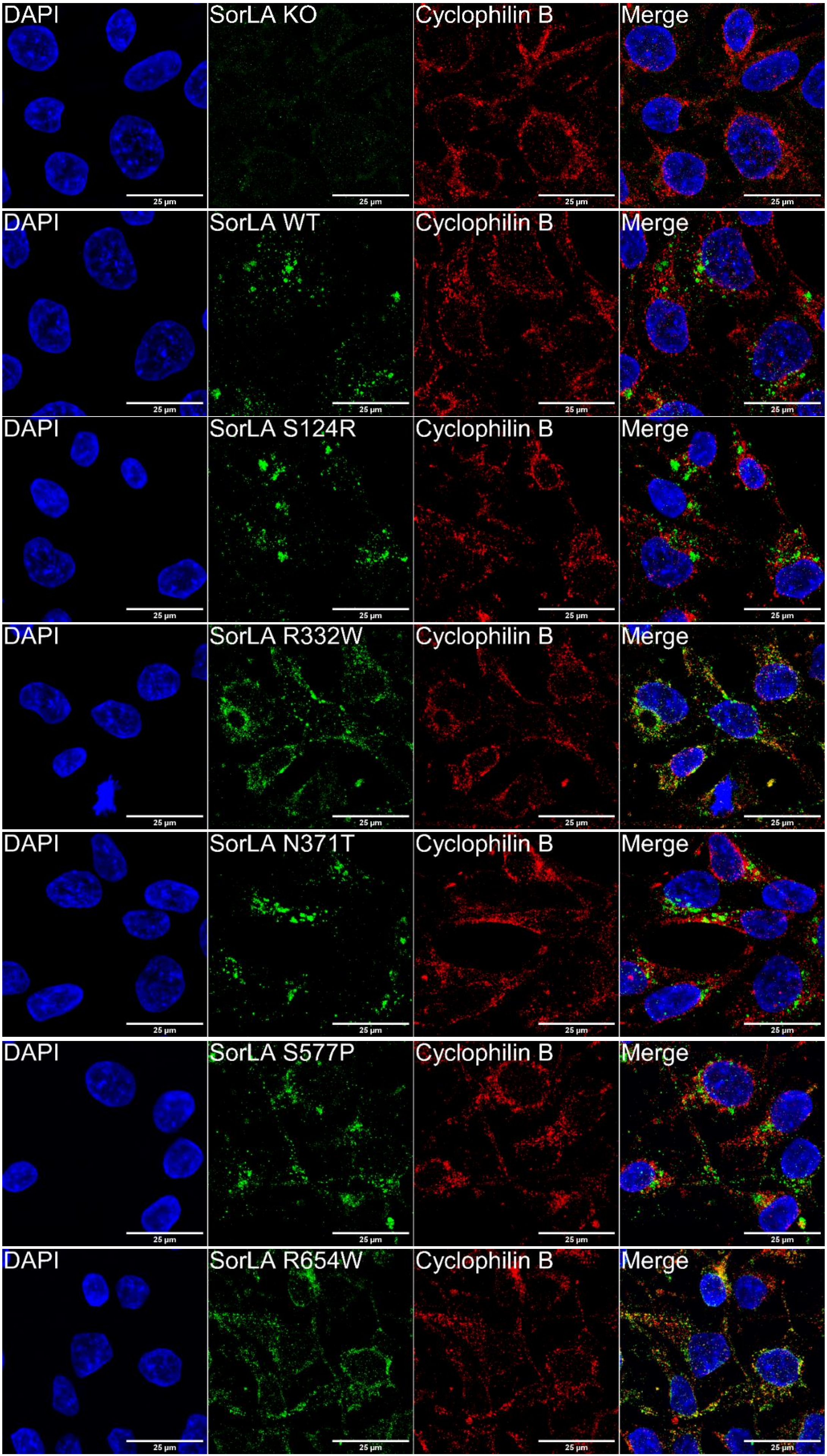
DAPI
SorLA KO
Cyclophilin B
Merge
SorLA WT
SorLA S124R
SorLA R332W
SorLA N371T
SorLA S577P
SorLA R654W
25 µm

Fig.S6

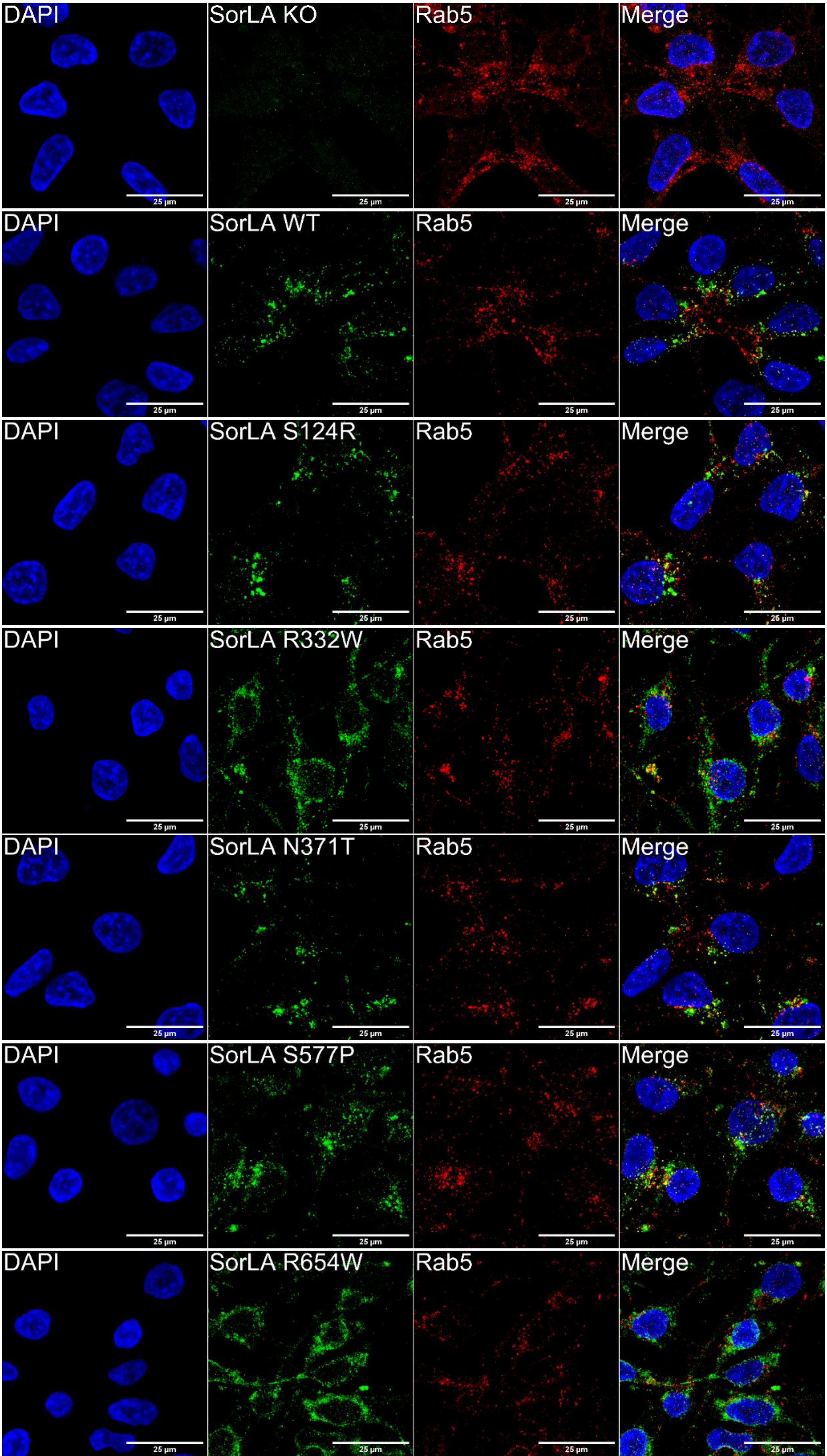

DAPI
SorLA KO
Rab5
Merge
SorLA WT
SorLA S124R
SorLA R332W
SorLA N371T
SorLA S577P
SorLA R654W
25 µm

Fig.S7

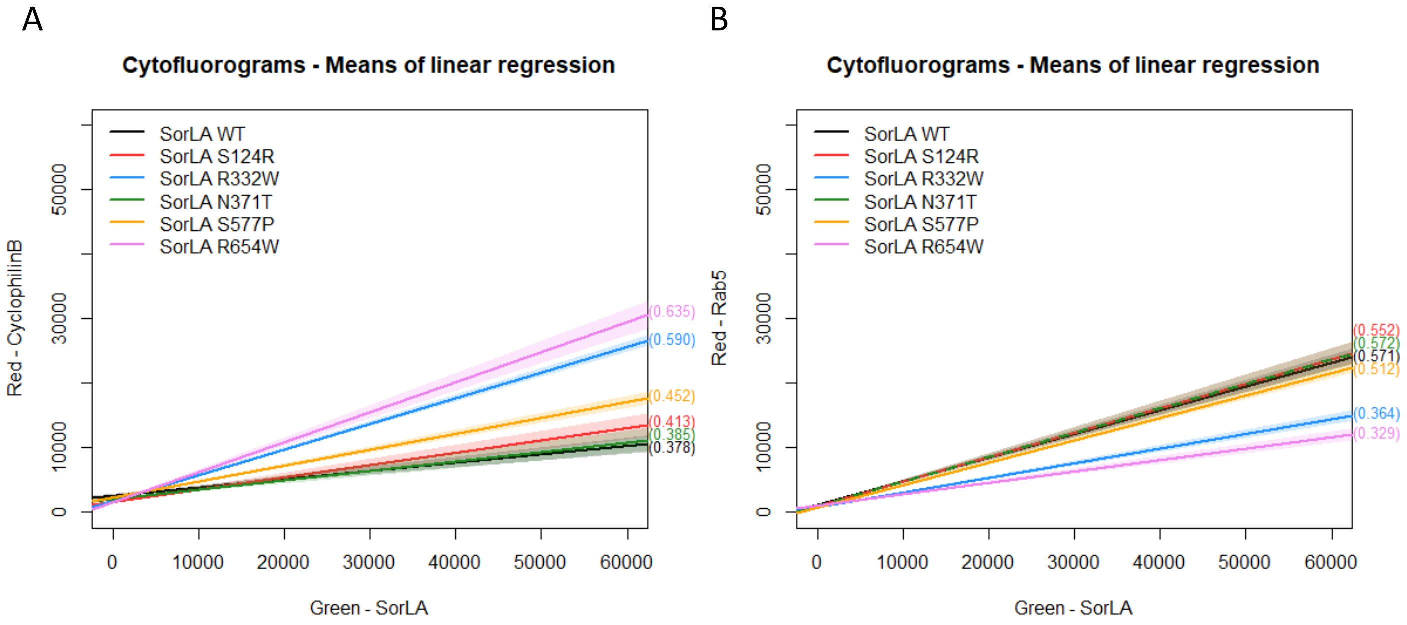
A
Cytofluorograms - Means of linear regression
SorLA WT
SorLA S124R
SorLA R332W
SorLA N371T
SorLA S577P
SorLA R654W
Red - CyclophilinB
Green - SorLA
0
10000
30000
50000
0
10000
20000
30000
40000
50000
60000
(0.635)
(0.590)
(0.452)
(0.413)
(0.385)
(0.378)
B
Cytofluorograms - Means of linear regression
SorLA WT
SorLA S124R
SorLA R332W
SorLA N371T
SorLA S577P
SorLA R654W
Red - Rab5
Green - SorLA
0
10000
30000
50000
0
10000
20000
30000
40000
50000
60000
(0.552)
(0.572)
(0.571)
(0.512)
(0.364)
(0.329)

Fig.S8
