# Supplementary material for: Impaired SorLA maturation and trafficking as a new mechanism for SORL1 missense variants in Alzheimer disease
Source: Acta Neuropathol Commun. 2021 Dec 18;9:196. doi: 10.1186/s40478-021-01294-4 (PMC8684260; doi:10.1186/s40478-021-01294-4)
Supplement: Supplementary file 2 — Additional file 2.: Legends of the supplementary figures. [file 40478_2021_1294_MOESM2_ESM.docx]

**Figure S1**: Representation of SorLA-^FL^ mature/immature ratio. For each variant, quantification was performed on two independent replicates. Practically, for each lane, the intensity of the upper and lower bands was quantified using the Genetools software, and the ratio upper / lower band was calculated. The mean ratio of the WT samples (two independent samples per blot) was arbitrarily set to 1, and the ratios of the variants were reported to the WT samples migrated on the same blot. On the graph are represented the quantifications from the two independent replicates for each variant, as well as the quantifications of all WT samples. The 15 variants selected for further analyses correspond to the variants with the lowest ratios, on the left of the line.

**Figure S2: Western blot analyses of SorLA^759^ and SorLA^2131^ proteins expressed in HEK293 cells.**

The SorLA^759^ (A) and SorLA^2131^ (B) proteins secreted into the cellular medium (Secreted) and the corresponding cell lysate (Intracellular) were analyzed by immunoblotting using an antibody specific to the N-terminus of human SorLA protein. For each variation, the patterns were confirmed in at least 4 independent replicates. Representative blots are presented. Note that the R654W and R729W variants showed an increase of their steady-state level in the cellular lysates compared to the wild-type SorLA^759^ construct. In most cases, proteins with transport defects are degraded by the ERAD/EGAD quality control system, suggesting that these mutant proteins were less effectively degraded by the quality control systems. The molecular masses of marker proteins in kDa are shown on the left.

**Figure S3: Sanger sequencing analysis of CRISPR/Cas9-edited hiPSC**

For each mutation, the electropherogram showing the insertion of the missense point mutation (on the bottom) is compared to the wild-type sequence (on the top). The nucleotide change on the cDNA and the corresponding amino acid change on the protein are indicated.

**Figure S4: Maturation defective SorLA variants display reduced cell-surface levels of SorLA protein in CRISPR/Cas9-edited hiPSC**

Surface biotinylation experiments to examine cell-surface levels of SorLA proteins in wild-type or S124R, R332W, N371T, S577P, R654W SorLA CRISPR/Cas9-edited hiPSC. Total lysates (Total) and biotinylated fraction**s** (Surface) were analyzed by immunoblotting using an anti-SorLA antibody. FUS, an intracellular protein, was used as an internal control. The absence of FUS in the biotinylated fraction demonstrates the integrity of the plasma membrane during the biotinylation experiment. Representative blots are presented. Immature core-glycosylated and mature complex-glycosylated SorLA are indicated with solid and empty arrowheads, respectively. The molecular masses of marker proteins in kDa are shown on the left.

**Figure S5: Sequencing and protein analysis of *SORL1* KO hiPSCs**

*SORL1* knock-out clones were selected during the screening of the clones carrying the R332W variant (see guide RNAs on Table S3). We chose clones carrying a homozygous indel, each consisting on an insertion of 1 nucleotide. (A) Sanger sequencing of the clones. The nucleotide insertion on cDNA, the corresponding amino acid change on the protein and the position of the premature stop codon are indicated. (B) Western blot showing the absence of SorLA staining in both *SORL1* KO clones, compared to wild-type (WT) clones. For each clone, two independent replicates were analyzed.

**Figure S6: Retention of SorLA maturation defective variants in the endoplasmic reticulum**

Wild-type and KO, S124R, R332W, N371T, S577P, R654W SorLA CRISPR/Cas9-edited hiPSC lines were double-labelled against SorLA (in green) and Cyclophilin B (in red). In blue, DAPI counter-staining.

**Figure S7: Decreased level of SorLA maturation defective variants in early endosomes**

Wild-type and KO, S124R, R332W, N371T, S577P, R654W SorLA CRISPR/Cas9-edited hiPSC lines were double-labelled against SorLA (in green) and Rab5 (in red). In blue, DAPI counter-staining.

**Figure S8: Colocalization analysis of SorLA maturation defective variants**

(A, B) Scatter plots representing for each SorLA genotype the mean (and its 95% confidence interval, represented by a halo) of linear regressions derived from corresponding two-dimensionnal cytofluorograms (not displayed to gain in visibility). Cytofluorograms depicted the distribution of pixels in two-color images according to their fluorescence intensity in the green channel (SorLA, x-axis) and in the red channel (cyclophilin *B* (A) or Rab5 (B); y-axis). For colocalisation analyses, more than ten images were acquired per co-labelling, leading to the genesis of more than ten cytofluorograms. The color code assigned to each genotype is the same as in Fig. 7*.* A line coinciding the first bisector indicates a perfect co-localisation. The numbers between brackets refer to the means of the M1 Manders’ coefficients calculated from the corresponding linear regressions. A shift of the linear regression from the first bisector is indicative of a decreased overlap and results in a smaller M1 coefficient. More the regression line deviates from the first bisector, more the overlap will be the M1 coefficient small.
