# Supplementary material for: Impaired SorLA maturation and trafficking as a new mechanism for SORL1 missense variants in Alzheimer disease
Source: Acta Neuropathol Commun. 2021 Dec 18;9:196. doi: 10.1186/s40478-021-01294-4 (PMC8684260; doi:10.1186/s40478-021-01294-4)
Supplement: Supplementary file 7 — Additional file 7: Table S5: Predicted results obtained with the Dynamut Server upon mutation. Mutations corresponding to maturation-defective proteins are colored in red. No correlation between impaired maturation and missense variants was observed. Indeed S114R, R332W and R654W were predicted to result in more stable structures (positive ΔΔG) while G543E, S654G, S577P and R729W were associated with a decrease of stability (negative ΔΔG). [file 40478_2021_1294_MOESM7_ESM.docx]

| **Variants** | ΔΔG (kcal/mol) | | ΔΔG (kcal/mol) | | ΔΔS_vib_ (kcal/mol)  **2** | |
| --- | --- | --- | --- | --- | --- | --- |
|  | **DynaMut** | | **ENCoM** | | **ENCoM** | |
| S114R | 0,326 | Stabilizing | 0,253 | Stabilizing | -0,316 | Decreased molecular flexibility |
| S124R | 0,969 | Stabilizing | 0,648 | Stabilizing | -0,81 | Decreased molecular flexibility |
| D140N | -0,622 | Destabilizing | -0,219 | Destabilizing | 0,273 | Increased molecular flexibility |
| Y141C | -1,108 | Destabilizing | -1,055 | Destabilizing | 1,318 | Increased molecular flexibility |
| R332W | 0,994 | Stabilizing | 0,438 | Stabilizing | -0,547 | Decreased molecular flexibility |
| N371T | -0,509 | Destabilizing | -0,098 | Destabilizing | 0,123 | Increased molecular flexibility |
| C473S | 0,618 | Stabilizing | -0,045 | Destabilizing | 0,057 | Increased molecular flexibility |
| G511R | 0,136 | Stabilizing | 0,244 | Stabilizing | -0,305 | Decreased molecular flexibility |
| G543E | -0,484 | Destabilizing | 0,756 | Destabilizing | -0,945 | Decreased molecular flexibility |
| S564G | -0,588 | Destabilizing | -0,631 | Destabilizing | 0,789 | Increased molecular flexibility |
| S577P | -0,411 | Destabilizing | -0,183 | Destabilizing | 0,229 | Increased molecular flexibility |
| S602L | 0,805 | Stabilizing | 0,116 | Stabilizing | -0,144 | Decreased molecular flexibility |
| R654W | 0,401 | Stabilizing | 0,505 | Stabilizing | -0,631 | Decreased molecular flexibility |
| N674S | -0,271 | Destabilizing | -0,047 | Destabilizing | 0,059 | Increased molecular flexibility |
| R729W | -0,083 | Destabilizing | -0,077 | Destabilizing | 0,096 | Increased molecular flexibility |
